# Meeting Report: “Metagenomics, Metadata and Meta-analysis” (M3) Workshop at the Pacific Symposium on Biocomputing 2010

**DOI:** 10.4056/sigs.802738

**Published:** 2010-06-15

**Authors:** Lynette Hirschman, Peter Sterk, Dawn Field, John Wooley, Guy Cochrane, Jack Gilbert, Eugene Kolker, Nikos Kyrpides, Folker Meyer, Ilene Mizrachi, Yasukazu Nakamura, Susanna-Assunta Sansone, Lynn Schriml, Tatiana Tatusova, Owen White, Pelin Yilmaz

**Affiliations:** 1 Information Technology Center, The MITRE Corporation, Bedford, MA, USA; 2 NERC Center for Ecology and Hydrology, Oxford, OX1 3SR, UK; 3 Wellcome Trust Sanger Institute, Hinxton, Cambridge, UK; 4 University of California San Diego, La Jolla, CA, USA; 5 European Molecular Biology Laboratory (EMBL), European Bioinformatics Institute (EBI), Hinxton, Cambridge, UK; 6 Plymouth Marine Laboratory (PML), Plymouth, UK; 7 Seattle Children’s Hospital, Seattle, WA, USA; 8 Genome Biology Program, DOE Joint Genome Institute, Walnut Creek, California, USA; 9 Argonne National Laboratory, 9700 South Cass Avenue, Argonne, IL 60439, USA; 10 National Center for Biotechnology Information, National Library of Medicine, National Institutes of Health, Bethesda, MD, USA; 11 DNA Data Bank of Japan, National Institute of Genetics, Yata, Mishima, Japan; 12 University of Maryland School of Medicine, Baltimore, MD, USA; 13 Microbial Genomics Group, Max Planck Institute for Marine Microbiology & Jacobs University Bremen, Bremen, Germany

## Abstract

This report summarizes the M3 Workshop held at the January 2010 Pacific Symposium on Biocomputing. The workshop, organized by Genomic Standards Consortium members, included five contributed talks, a series of short presentations from stakeholders in the genomics standards community, a poster session, and, in the evening, an open discussion session to review current projects and examine future directions for the GSC and its stakeholders.

## Introduction

The M3 Workshop at the Pacific Symposium on Biocomputing (PSB) 2010 was organized by members of the Genomic Standards Consortium to continue the outreach by the GSC to the broader multi-omics community and to the computational biology community. The workshop was a follow-on to two successful workshops held during the second half of 2009: the International Conference on Intelligent Systems for Molecular Biology (ISMB) Metagenomics, Metadata and MetaAnalysis (M3) Special Interest Group (SIG) [1], and the M5 (Metagenomics, Metadata, MetaAnalysis, Models, and Metainfrastructure) workshop held in conjunction with the Supercomputing ’09 (SC09) conference, Portland, OR, United States.

PSB serves as a meeting ground to explore topical issues of interest to a cross section of the computational biology community. In addition to the M3 Workshop, this 2010 PSB meeting included six sessions and two other workshops:

- Computational Challenges in Comparative Genomics
- Computational studies of non-coding RNAs
- Dynamics of Biological Networks
- Multi-resolution Modeling of Biological Macromolecules
- Personal Genomics
- Reverse Engineering and Synthesis of Biomolecular Systems
- *In silico* Biology Workshop
- GPD-Rxn Workshop: Genotype-Phenotype-Drug Relationship Extraction from Text

## Background

The Genomic Standards Consortium (GSC) organized this workshop as part of its goal to create richer descriptions for the collection of genomes and metagenomes through the development of standards and tools for supporting compliance and exchange of contextual information [2]. Established in September 2005, this international community includes representatives from the International Nucleotide Sequence Database Collaboration (INSDC), major genome sequencing centers, bioinformatics centers and a range of research institutions.

The rapid pace of genomic and metagenomic sequencing projects [3], which now include studies of microbiomes, will only increase as the use of ultra-high-throughput sequencing methods becomes more commonplace. It is clear that we need new standards to capture additional contextual data as well as tools to support its use in downstream computational analyses. It is also clear that these standards will be vital to exploring the complex interactions that take place in communities – both microbial communities, such as those sampled in marine environments, and host-microbial communities, such as those now being sampled in the Human Microbiome Project.

The GSC has been responsible for promulgating the MIGS/MIMS standard (Minimal Information about Genomic/Metagenomics Sequences) [3], and, at the 8th GSC workshop in September 2009, a new standard MIENS (Minimal Information about an ENvironmental Sequence) [4]. These standards are being incorporated into the INSDC (International Nucleotide Sequence Database Collaboration) as part of a new “structured comment field”. This development was explored in a panel session that was part of the workshop, involving representatives from DDBJ, EMBL and GenBank.

As one of its activities, the GSC has launched a new electronic journal SIGS (Standards in Genomic Sciences (http://standardsingenomics.org/) in order to provide an open-access publication for the rapid dissemination of both genome and metagenome reports compliant with the MIGS/MIMS standards; the first three issues have included “Short Genome Reports” on 32 sequenced bacterial genomes.

The M3 Workshop at PSB 2010 built directly on the past GSC workshops and the ISMB SIG [1]. Its focus was on comparative studies of (meta)genomes that bring these sequences into "context" (i.e., by geolocation, habitat, organism phenotype, etc). A recent paper published in PNAS illustrates the power of this approach [5]. It reports a study aimed at elucidating the relationships between metabolic pathways and environmental parameters in microbial communities using the data and metadata from the Global Ocean Survey (GOS), an earlier landmark paper in the history of the field of metagenomics [6]. The kick-off of the Human Microbiome Project and the resulting data sets will open enormous new possibilities for the coordinated integration of contextualized metagenomes.

## M3 Workshop Structure

The workshop goal was to attract experimentalists and computational researchers making "next-generation" use of contextual metadata. The workshop was divided into two parts – a set of contributed talks to highlight specific research activities, and a panel of leaders in the metagenomics community who discussed the broad issues related to generation of metagenomics data, metadata standards and tools to support the meta-analysis. In addition, the workshop included a poster session to highlight recent advances related to the M3 goals and GSC activities.

### Contributed Talks

The contributed talks covered the three “M”s:

**Metagenomics**
- *Using 100 years of data to contextualize metagenomics in the Western English Channel.* Jack Gilbert, Plymouth Marine Laboratory, UK
- *Metagenomics reveals functional shifts in the bovine rumen microbiota composition with propionate intake.* Michael E. Sparks, Animal and Natural Resources Institute, USDA, Agricultural Research Service, Beltsville, USA

**Metadata**
- *Gemina: Ontology and metadata standards development provide core of infectious pathogen surveillance and geospatial tool.* Lynn Schriml, University of Maryland School of Medicine, Baltimore, USA

**Meta-analysis**
- *Comparative Microbial genomics of resistance genes in* *Staphylococcus aureus*. Anja Stausgaard, The Technical University of Denmark, 2800 Kgs. Lyngby, Denmark
- *Accurate taxonomic assignment of short pyrosequencing reads*. Jose Clemente, Center for Information Biology and DNA Databank of Japan, National Institute of Genetics, Mishima, Japan.

The first two talks (Gilbert, Sparks) described comparative metagenomic studies that demonstrated the power provided by data measured (e.g. geographic location, salinity, temperature, or pH) and curated (e.g., habitat or host) using appropriate metadata standards. The third talk by Schriml described a new set of curated metadata standards that aided in the integration and inter-operability of disparate datasets, drawing on GSC sponsored work on the Environmental Ontology EnvO. The final two talks demonstrated the power of meta-analysis: Stausgaard used a comparative genomics approach to identify and analyze resistance genes in *Staphylococcus aureus*); Clemente looked at taxonomic assignment of sequences of short read-length, a significant hurdle for metagenome annotation from ultra-high-throughput sequencing platforms such as Illumina and SOLiD

The contributed talks were followed by flash presentations for posters, which were available during the break as well as later, during the main conference.

### Panel Discussion

The panel began with a set of reports from the INSDC members: Cochrane for EBI, Nakamura for DDBJ, Mizrachi for NCBI. Cochrane reported on the inclusion of structured comments and support for the new MIENS standard. This triggered some discussion about validation of entries for the structured comments fields, and the feasibility of using ontologies or controlled vocabularies in these fields.

The second part of the panel included reports from RefSeq [7] (Tatusova), the ISA Infrastructure [8] (Sansone), GEBA [9] and GOLD [10] (Kyrpides), CAMERA [11] (Grethe), the recent M5 workshop, a new approach to consensus annotation (White), and computational infrastructure needs (Meyer).

### Evening Open Discussion

The evening session drew over 20 people for a lively discussion. One topic was how to identify other venues that might be productive, in terms of "getting out the word" and attracting new participants. Suggestions included the International Symposium for Microbial Ecology (ISME) meeting in August 22-27^th^ in Seattle. This had now led to the inclusion of a GSC round table discussion at this meeting on Monday the 23^rd^ August 2010. There was discussion of both previous meetings in which the GSC was invited to participate, including the 109^th^ General Meeting of the American Society for Microbiology (ASM), the Argonne Soils Workshop and SC09, as well as upcoming GSC sponsored events including the M3 and BioSharing SIG at ISMB 2010, July 9-10 in Boston, and the GSC9 meeting at JCVI April 28-30^th^ 2010 in Rockville. In addition, Nikos Kyrpides made a plea for the GSC to reach beyond the microbial community to include the plant genome community as well as many of the model organism groups.

There was discussion about a different meaning of "standards" that might serve as a kind of "Consumer Reports" model for comparing and contrasting different tools that could be used for various parts of the annotation pipeline. There was discussion about whether GSC might provide or encourage clear descriptions of current annotation pipelines, building on a meeting before SC09 that discussed capture and exchange of workflows. Another idea was to identify bottlenecks where current methods do not scale; these could perhaps be posed as "challenges" for the computational biology community. There was discussion about whether GSC might put together some gold standard data sets in order to support some kind of CASP-like (Critical Assessment of protein Structure Prediction [12]) or BioCreative-like (Critical Assessment of Information Extraction for Biology [13]) competition.

There was discussion about how the GSC could interact with industry. Several people commented that many of the sequencing companies are hoping that the research community will develop algorithms to handle the flood of data coming out of the next generation sequencers. This might present an opportunity to interact with the commercial sector in a cooperative mode. Jack Gilbert reported that he was already raising money from industry for GSC9 (this resulted in an inclusion of an industry panel at the GSC9 meeting – successfully integrating industrial partners in to the GSC vision); also Folker Meyer reported that Amazon has offered up a computing environment for large scale experiments.

There was a brief discussion of places where controlled vocabularies and text mining might be useful - this was a continuation of discussion from the panel session, related to the use of structured comments and validation of the content of a field. There was discussion about the trade-offs of using of a controlled vocabulary - the pluses are that the values can be validated and may be more readily "computable" (if using an ontology); the cons are that this requires community buy in - and must not be allowed to create any additional obstacles to data entry. Apparently there is still quite limited buy-in for researchers to deposit richly annotated data.

## Conclusions

The organizers felt that this had been a successful workshop. It was well-attended (around 40 participants during the main session, and about half that number in the evening session). The GSC presence at PSB enabled a number of informal side-discussions and exchanges that would not have happened otherwise.
